# How herpes is assembled

**DOI:** 10.7554/eLife.106419

**Published:** 2025-03-17

**Authors:** Dawid S Zyla

**Affiliations:** 1 La Jolla Institute for Immunology La Jolla United States

**Keywords:** viral envelope, Herpes simplex, replication, proteins, vesicles, temporary envelope, Viruses

## Abstract

A combination of imaging techniques reveals how herpes simplex virus type 1 assembles within infected cells, highlighting the roles of essential viral proteins in viral assembly and exit.

**Related research article** Nahas KL, Connor V, Wijesinghe KJ, Barrow HG, Dobbie IM, Harkiolaki M, Graham SC, Crump CM. 2025. Applying 3D correlative structured illumination microscopy and X-ray tomography to characterise herpes simplex virus-1 morphogenesis. *eLife* **14**:RP105209. doi: 10.7554/eLife.105209.

Viruses are remarkably diverse pathogens that can infect all life forms. Despite their differences in shape and size, they share a common goal: to hijack a host cell’s machinery and use it to replicate and spread. Some viruses rely on only a handful of proteins to complete their life cycle, while others encode dozens or even hundreds. Understanding the roles of these viral proteins is crucial for developing effective antiviral therapies.

Herpes viruses are among the most widespread human viruses, establishing lifelong infections by evading the immune system and remaining dormant in cells (Schleiss, 2009; Verzosa et al., 2021). While some cause relatively mild illnesses, such as cold sores, chickenpox or shingles, others pose serious risks to immunocompromised individuals and have also been linked to certain cancers (Sehrawat et al., 2018; Goncalves et al., 2017).

Herpes simplex virus type 1 (HSV-1) is a well-known member of this family and is primarily responsible for oral and genital sores. It has a large DNA genome of over 150,000 base pairs, encoding more than 70 proteins that govern different stages of its life cycle, including replication and assembly (Davison, 2011; Kelly et al., 2009; Kounatidis et al., 2020). Although studying individual viral proteins provides valuable insights, it does not always reveal how they interact collectively during the viral life cycle. A more holistic approach is often required to fully understand the viral assembly process (Watanabe et al., 2024; Kreysing et al., 2025).

Now, in eLife, Colin Crump, Stephen Graham, Maria Harkiolaki and colleagues – including Kamal Nahas (University of Cambridge and the Diamond Light Source) as first author – report new insights into how HSV-1 assembles within infected cells (Nahas et al., 2025). The researchers, who are based at Cambridge, the University of Warwick, the Diamond Light Source and other institutions in the UK, Sri Lanka and the US, used an innovative combination of imaging techniques to study nine HSV-1 variants, each missing a single viral protein essential for assembly but with previously unknown roles in the process. Systematically removing these proteins clarified their specific functions in viral assembly, providing new insights into the mechanisms governing herpes virus maturation.

To visualize these events, Nahas et al. employed cryo-structured illumination microscopy (cryo-SIM) and cryo-soft X-ray tomography (cryo-SXT) – two powerful imaging techniques that allow viruses to be studied in isolated live cells (Kounatidis et al., 2020). Cryo-SIM uses patterned light and advanced algorithms to obtain super-resolution images of frozen biological samples. In contrast, cryo-SXT uses low-energy X-rays to create 3D reconstructions of the internal structures of frozen cells, revealing their organization. Unlike traditional electron microscopy techniques, which deliver high-resolution snapshots of small regions, these methods offer a broader view of infected cells, enabling a more comprehensive understanding of the viral assembly process.

Nahas et al. uncovered several key findings. First, they revealed that proteins such as pUL16, pUL21, pUL34, VP16 and pUS3 were critical for HSV-1 to emerge from the host nucleus. This crucial step happens early in the viral life cycle once DNA has been replicated and enclosed within a protein capsid (Figure 1).

**Figure 1.**
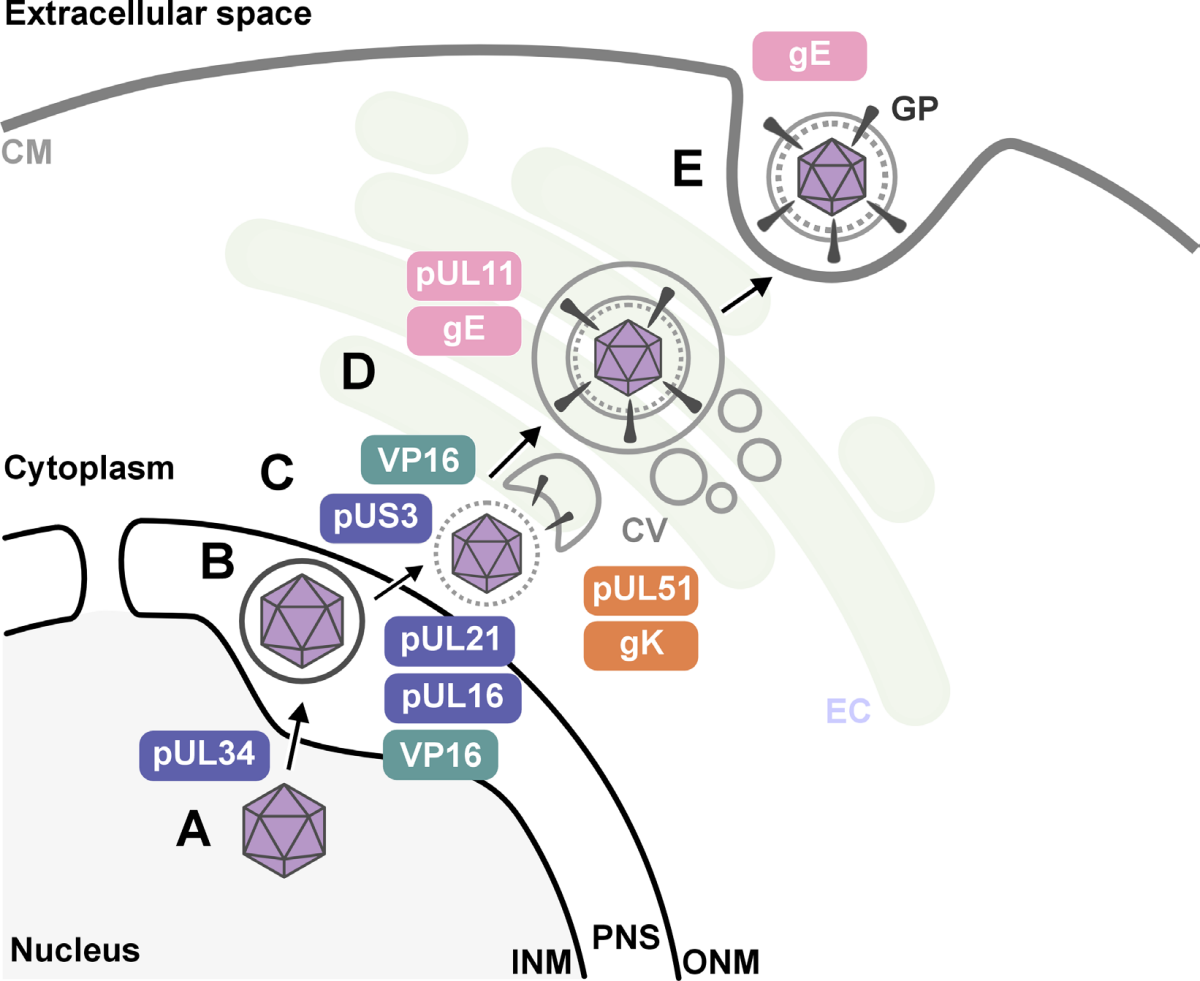
The key stages of Herpes simplex virus 1 (HSV-1) assembly and the roles of specific viral proteins, based on advanced imaging data. (**A; lower left**) In the host cell nucleus, HSV-1 DNA is replicated, and a protein capsid that encloses the viral genome is assembled. These capsids (purple hexagons) bind to pUL34, a component of the nuclear egress complex (NEC) and move towards the inner nuclear membrane of the host cell (INM). The capsid-protein complexes then bud into the perinuclear space (PNS) between the inner and outer membrane of the cell nucleus, where they acquire a temporary envelope from the INM (depicted as a circle around the hexagon). (**B**) The viral complexes then fuse with the outer nuclear membrane (ONM), where they shed their temporary envelope and are released into the cytoplasm. The viral kinase pUS3 regulates the NEC and thus influences the efficiency of the capsids transit through the nuclear envelope. (**C**) Once in the cytoplasm, VP16 binds to the capsid to stabilize it for further maturation. Then, pUL16 starts to form the tegument (gray dotted circle), a protein-rich layer that surrounds the capsid and connects it to the outer layer, the envelope, with pUL21 assisting in cytoplasmic transport of molecules between the two layers (**D**) VP16 delivers the capsids to the envelopment compartment (EC), where they associate with cytoplasmic vesicles (CV, gray circles) to form secondary envelopes with the help of pUL11, pUL51, gK and gE. (**E**) Finally, the mature virus particle is transported within the vesicle originating from the envelopment compartment, which then fuses with the cell membrane (CM) to release the mature virion with viral glycoproteins (GP) into the extracellular space.

Second, the research shed new light on how HSV-1 acquires its outer layer, known as the outer envelope, revealing that this step is more intricate than previously thought. The imaging studies revealed that the process involved a series of coordinated interactions between viral proteins – including pUL11, VP16, pUL51, gK and gE – and the membranes of intracellular vesicles that are part of the cell’s trans-Golgi network and endosomal pathways. Notably, the researchers observed that the viral envelope compresses just before the virus buds off the host cell, which may be essential for viral stability and infectivity.

Overall, the findings of Nahas et al. improve our understanding of how the HSV-1 virus is assembled and demonstrate the value of advanced imaging techniques in virology. These findings also raise new questions about the functions of other viral proteins and how they interact with host cell components. By uncovering key steps in the assembly process, this research could lead to the development of new antiviral strategies that target specific stages of viral maturation. The insights gained from HSV-1 may extend to other herpes viruses and provide new ways to study complex viral processes within the cell.
